# Sensitivity and Cellular Labelling Performance of Magnetically Fractionated SPIONs for Multimodal MRI/MPI Imaging

**DOI:** 10.3390/nano16161022

**Published:** 2026-08-18

**Authors:** Nicola Greco, Anita Conti, Arnaud Martino Capuzzo, Giusi Piccolantonio, Alessandro Negri, Mandy Ahlborg, Pascal Stagge, Eric Aderhold, Kerstin Lüdtke-Buzug, Ermanna Turano, Ilaria Scambi, Mauro Caprioli, Raffaella Mariotti, Pietro Bontempi, Pasquina Marzola

**Affiliations:** 1 Department of Engineering for Innovation Medicine, University of Verona, Strada le Grazie, 15, 37134 Verona, Italy; nicola.greco@univr.it (N.G.); anita.conti@univr.it (A.C.); arnaudmartino.capuzzo@univr.it (A.M.C.); giusi.piccolantonio@univr.it (G.P.); alessandro.negri@univr.it (A.N.);; 2 Institute of Medical Engineering, University of Luebeck, Ratzeburger Allee 160, 23562 Lübeck, Germany; mandy.ahlborg@imte.fraunhofer.de (M.A.); pascal.stagge@imte.fraunhofer.de (P.S.); eric.aderhold@imte.fraunhofer.de (E.A.); 3 Institute of Medical Engineering, University of Lübeck, 23562 Lübeck, Germany; kerstin.luedtkebuzug@uni-luebeck.de; 4 Department of Neuroscience, Biomedicine and Movement Sciences, University of Verona, 37134 Verona, Italy; ermanna.turano@univr.it (E.T.); ilaria.scambi@univr.it (I.S.); mauro.caprioli@univr.it (M.C.); raffaella.mariotti@univr.it (R.M.)

**Keywords:** SPION, MRI, MPI, labelled cells, labelled EVs

## Abstract

Magnetic Particle Imaging (MPI) detects superparamagnetic nanoparticles, enabling bimodal contrast with MRI. Resovist^®^/Ferucarbotran, still used in research, has clinical safety compatibility but suboptimal MPI performance due to many small 5 nm SPIO cores. Magnetic fractionation can enrich larger cores, improving MPI signal and supporting cellular imaging applications. To enable bimodal MRI/MPI and assess in vivo extracellular vesicle (EV) labelling, we characterized the imaging sensitivity and cell-labelling performance of VivoTrax, a commercial formulation similar to Resovist^®^, and VivoTrax Plus, obtained by magnetic fractionation. VivoTrax Plus showed higher MRI transverse relaxivity and MPI sensitivity than VivoTrax, and both formulations displayed low toxicity toward adipose-derived stem cells (ASCs). VivoTrax Plus allowed MRI detection of small cell numbers, around 100 cells, in agarose phantoms with greater sensitivity than VivoTrax. However, EVs of 30–150 nm isolated from ASCs labelled with VivoTrax Plus did not retain SPIONs, whereas EVs from VivoTrax-labelled ASCs did. Preliminary in vivo experiments in SOD-G93A mice showed MRI-detectable signal voids in the brain after intranasal EV administration, suggesting EV migration to lesioned areas. Overall, magnetic fractionation improves SPION imaging sensitivity but may alter relevant biological properties.

## 1. Introduction

The development of novel contrast agents has been a significant research focus for enhancing the sensitivity and specificity of magnetic resonance imaging (MRI) and, more recently, for magnetic particle imaging (MPI). Superparamagnetic iron oxide nanoparticles (SPIONs) have emerged as versatile tools in theranostics, particularly as MRI contrast agents, owing to their unique physicochemical properties and tunable magnetic behaviour [1,2]. Gadolinium-based chelates are traditional MRI contrast agents used in clinical settings; however, advanced applications of cellular and molecular imaging require enhancements in relaxivity and pharmacokinetic properties for improved efficacy. On the other hand, nanoparticle-based contrast agents, such as SPIONs, have been extensively studied and offer potential advantages, including low toxicity, good biocompatibility, biodegradability, and improved relaxivity properties [3,4]. Despite their promise, most nanoparticles (NPs) have not yet reached clinical application, underscoring the need for further research to address challenges related to toxicity, stability, and in vivo performance [5]. Various approaches, such as surface modification with biocompatible coatings such as dextran or peptide amphiphiles, have been explored to enhance their performance and safety for clinical use [6,7,8]. Among the nanoparticle-based contrast agents that have reached clinical approval, several superparamagnetic MRI contrast agents were commercialized in the early 2000s and introduced into clinical practice primarily as hepatospecific diagnostic agents. Nearly all of them were later withdrawn from the market due to an unfavourable cost–benefit ratio. Nonetheless, some agents continue to be produced and used for research purposes, such as Resovist^®^ (also known as Ferucarbotran or VivoTrax), and are now regarded with renewed interest given that new applications have been developed, ranging from advanced cellular imaging to emerging new imaging modalities.

Resovist^®^ is a carboxy-dextran cross-linked clustered SPIONs tracer commonly used in MPI, composed of small 4–6 nm iron oxide nanocrystals organized into a cluster. Technically, only a small percentage of nanocrystals are closely bound together into clusters that magnetically interact to produce a signal. The remaining iron oxide nanocrystals, which form most of the samples, are magnetically non-interacting and do not produce a significant MPI signal [9]. Resovist^®^ demonstrates improved dispersibility and longer circulation times than other commercial MRI contrast agents, making it a promising candidate for MRI and MPI applications [10,11]. In recent years, Resovist^®^ has been produced and commercialized for research purposes under the tradename of VivoTrax by Magnetic Insight, Alameda, CA, USA. VivoTrax Plus is a modified form of VivoTrax that has been engineered by magnetic fractionation to increase magnetically interacting cores, thereby enhancing its magnetic performance in MRI and MPI applications [9]. It is worth mentioning that commercialization of VivoTrax and VivoTrax Plus was discontinued only recently. Nevertheless, because Resovist^®^/Ferucarbotran is still produced and magnetic fractionation can be applied to other SPIONs, our results remain relevant to the field. While the improvement in magnetic performance obtained through magnetic fractionation has been previously described, its potential impact on the biological behaviour of the nanoparticles, particularly in cell labelling and extracellular vesicle loading, has not yet been systematically investigated.

Both VivoTrax and VivoTrax Plus act as T2-weighted MRI contrast agents, inducing local magnetic field inhomogeneities that reduce T2 and T2* relaxation times, producing hypointense (dark) signals and notable blooming effects [12,13]. Although VivoTrax and VivoTrax Plus share several features, they exhibit distinct relaxivity properties, which impact their performance as MRI/MPI contrast agents. Superparamagnetic NPs have been widely used to label stem cells for MRI tracking, owing to their high relaxivity and ability to induce signal voids in MRI. This strategy allows non-invasive monitoring of cell migration and therapeutic distribution, helps to determine whether stem cells reach the target site, and allows investigation into the potential therapeutic effects of stem cell-based therapy [12,14].

Stem cell-derived extracellular vesicles (EVs) have emerged as promising tools for the diagnosis and treatment of various diseases, including neurodegenerative disorders. These nanometre-sized membrane-bound vesicles can deliver bioactive macromolecules such as proteins, lipids, and nucleic acids, facilitating intercellular communication [15]. Stem cell-derived EVs have demonstrated therapeutic potential in several neurological conditions—including stroke, traumatic brain injury, Alzheimer’s disease, and Parkinson’s disease [16]—owing to their ability to cross the blood–brain barrier [17]. Notably, they offer several advantages over traditional cell-based therapies, including better biocompatibility, immunogenicity, and safety profiles [16]. However, challenges remain in optimizing the EV production, isolation, and delivery methods for clinical applications [18]. To enable non-invasive MRI tracking of therapeutic EVs, a commonly adopted strategy is to label the parental cells with SPIONs prior to EV isolation. Following endocytic uptake, SPIONs are trafficked through the endosomal pathway, enabling their incorporation into EVs during vesicle biogenesis. The resulting SPION-loaded EVs retain the biological properties of native EVs while becoming detectable by MRI, allowing non-invasive monitoring of their biodistribution and homing in vivo. In addition, it remains unclear whether the physicochemical modifications of NPs influence the efficiency with which SPIONs are transferred from parental stem cells into EVs.

Therefore, this study was designed not only to compare the MRI and MPI performance of VivoTrax and VivoTrax Plus, but also to investigate whether magnetic fractionation influences their physicochemical properties, imaging performance and biocompatibility, including stem cell labelling and the subsequent incorporation of SPIONs into ASC-derived extracellular vesicles. Additionally, we proposes their application for adipose stem cell (ASC) labelling and for generating MRI-visible EVs, focusing on imaging characteristics to track therapeutic agents in real-time in preclinical ALS models. By examining these SPION formulations, this study aimed to elucidate their respective advantages and limitations, contributing to the optimization of nanoparticle-based contrast agents for future imaging applications.

## 2. Materials and Methods

### 2.1. Physicochemical Characterization of Nanoparticles

Commercial VivoTrax and VivoTrax Plus (Dextran Magnetite Iron oxide γ-Fe_2_O_3_, core diameter 4.2 nm and hydrodynamic diameter particle size 62 nm, stock solution 5.30–5.86 mg/mL) were purchased from Magnetic Insight (Alameda, CA, USA). VivoTrax corresponds to Ferucarbotran (Resovist^®^) and is licensed for distribution and preclinical MPI applications in the US.

Dynamic light scattering (DLS) was used to measure the hydrodynamic size and polydispersity index of the NPs, using a Zetasizer Nano-ZS instrument (Malvern Instruments, Malvern, UK) calibrated to a wavelength of 633 nm and equipped with a 173° backscattering detector. Measurements were conducted at 25 °C, with each sample undergoing six to seven acquisitions at 10 s interval. NPs were dispersed in water, phosphate-buffered saline (PBS), HEPES, and Dulbecco’s modified Eagle’s medium-high glucose GlutaMAX supplement (DMEM-HG). All reagents for cell cultures were purchased from GIBCO Life Technology (Milan, Italy). The zeta potential was measured only in aqueous solution.

Nanoparticle tracking analysis (NTA) was performed to analyze the particle dimensions and concentrations using a NanoSight NS300 (Malvern Panalytical, Malvern, UK) equipped with a 488 nm laser, and a syringe pump system with a pump speed of 20 µL/s. Data acquisition and processing were conducted using NTA 3.4 software (Malvern Panalytical).

Transmission Electron Microscopy (TEM) imaging was performed using a Morgagni 268D (Fei Company-Philips, Eindhoven, The Netherlands) operating at 80 kV and equipped with a MegaView G3 camera (EMSIS Gmbh, Munster, Germany). The NPs were deposited on a Formvar/Carbon 300 Mesh Copper (Electron Microscopy Sciences, Hatfield, PA, USA) and imaged.

To evaluate MRI relaxivity, NPs were dissolved in a range of biological and buffer media: deionized water, PBS (no Ca^2+^ or Mg^2+^), HEPES (10 mM), HEPES-PBS mixture, complete culture media (DMEM + 10%FBS), and artificial cerebrospinal fluid (aCSF). aCSF was prepared using the following composition: NaCl 125 mM, NaHCO_3_ 26 mM, NaH_2_PO_4_ 1.25 mM, MgCl_2_ 1 mM, KCl 2.5 mM, CaCl_2_ 2 mM, Glucose 10 mM, Ascorbic Acid 1 mM. During preparation, the solution was equilibrated with carbogen (95% O_2_/5% CO_2_) to maintain the bicarbonate/CO_2_ buffering system and stabilize the pH to physiological conditions (7.2–7.4). The iron concentration in the solutions ranged from 0.5 to 0.06 mM Fe. MRI acquisitions were performed using a Bruker Tomograph operating at 7.0 T controlled by Paravision software, version 6.0.1 (Bruker BioSpin, Ettlingen, Germany).

The longitudinal relaxation times (T1) of these solutions were measured using a saturation recovery approach with a fast spin-echo MRI pulse sequence (RARE: Rapid Acquisition with Relaxation Enhancement), with the following major parameters: minimum Repetition Time (TR) = 125 ms, maximum TR = 15,000 ms, Echo Time (TE) = 7 ms, Number of TR = 15, Field of View (FOV) = 4.5 × 3.5 cm^2^, matrix (MTX) = 128 × 128, slice thickness = 1.5 mm, number of slices = 5, and Number of Excitations (NEX) = 1. T1 relaxation maps were calculated using pixel-wise fitting of a saturation recovery model for the image series.

Transverse relaxation times (T2) were measured using a Multi Slice Multi Echo (MSME) pulse sequence, with the following parameters: TR = 10,000 ms, 64 evenly spaced echo times, minimum TE = 7 ms, maximum TE = 448 ms, FOV = 4.5 × 3.5 cm^2^, MTX = 128 × 128, slice thickness = 1.5 mm, number of slices = 5, and NEX = 1. T2 decay maps were calculated using pixel-wise fittings of the mono-exponential decay to the image series. Relaxation rates R1 = 1/T1 and R2 = 1/T2 were calculated from T1 and T2 maps in the regions of interest (ROIs) covering the individual sample tubes. The NPs relaxivity was then estimated through linear fitting of R1 and R2 values versus the iron concentration [mM].

Magnetic Particle Spectroscopy (MPS) measurements were conducted using a custom-built device [19]. The excitation field was applied at a frequency of 25 kHz with an amplitude of 20 mT. For each measurement, the signals were averaged over 1000 repetitions to improve the signal-to-noise ratio. A sample volume of 10 µL was used for this study. The samples were prepared from a stock solution with a concentration of 5500 µg Fe/mL and diluted to 200, 12.5, and 3.1 µg Fe/mL.

Magnetic Particle Imaging (MPI) experiments were conducted using a custom-built field-free line (FFL) system [20]. The drive field amplitude was 20 mT at a frequency of 25 kHz and the focus field amplitude was 10 mT at a frequency of 200 Hz. FFL rotation was set to 1 Hz. Experiments with VivoTraxPlus were performed at concentrations of 200 and 50 µg Fe/mL. The phantom consists of a glass capillary with an inner diameter of 0.45 mm. The imaging plan is orthogonal to the tube; thus, a circle with diameter 0.45 mm is to be expected in the images. Image reconstruction was performed using MPIReco.jl (v0.9.6) [21].

### 2.2. Interaction of Nanoparticles with ASC

Murine ASCs were obtained from inguinal adipose tissues of 8-/12-week-old C57Bl/6 mice (Charles River Laboratories, Italy), as previously described [22,23]. In vivo experiments were conducted in accordance with experimental guidelines approved by the University of Verona Committee on Animal Research (Centro Interdipartimentale di Servizio alla Ricerca Sperimentale) and by the Italian Ministry of Health (protocol #56DC9.72). After isolation, the cells were cultured in DMEM-HG supplemented with 10% fetal bovine serum (FBS), 100 U/mL penicillin, and 100 µg/mL streptomycin (all from GIBCO Life Technologies, Milan, Italy). Cells were maintained in a humidified incubator with 95% air and 5% CO_2_ at 37 °C.

The 3-(4, 5-dimethylthiazol-2-yl)-2, 5-diphenyltetrazolium bromide (MTT) assay was performed to assess cell viability for cytotoxicity testing after VivoTrax and VivoTrax Plus treatments. Briefly, 3 × 10^4^ ASCs were seeded in 96-well plates with 100 µL cell culture medium. The NPs concentration was set at 200, 100, 50, 25, 12.5, and 6.2 µg Fe/mL and two time points were established (24 and 48 h). After incubation, the cells were washed twice with PBS and MTT solution was added at the concentrations indicated by the manufacturer’s instructions. After 4 h of incubation, the absorbance was recorded using a spectrophotometer microplate reader (Infinite M-Plex, Infinite M-Plex, Tecan, Männedorf, Switzerland) at 570 nm (MTT-specific absorbance) and 660 nm (to eliminate background signals for all non-specific absorbance).

ASCs (2.5 × 10^5^) were seeded per well onto 13 mm sterilized coverslips. The cells were then treated with 500 µL solutions of NPs diluted in culture media at concentrations of 200, 50, and 6.2 µg Fe/mL for 24 h. The cells were washed twice with PBS and fixed with 4% paraformaldehyde (PFA) for 10 min. To visually examine iron uptake, histological analysis was performed using Prussian blue staining and counterstaining with hematoxylin and eosin. The internalization of the NPs was subsequently assessed using an Olympus APXVIEW APX100 optical microscope (Olympus Italia S.r.l., Segrate, MI, Italy) for digital image acquisition.

To elucidate the internalization mechanism and ultrastructural localization of NPs, electron microscopy was performed on the ASC monolayer. ASCs were seeded at 2.5 × 10^5^ per well onto 13 mm sterilized coverslips and incubated with 200 µg Fe/mL of NPs for 24 h. The cells were washed twice with PBS, fixed in 2% glutaraldehyde in Sorensen buffer (pH 7.4) for 1 h, and post-fixed in 1% osmium tetroxide (OsO4) diluted in 0.2 M K_3_Fe(CN)_6_ for 30 min. The samples were dehydrated in graded concentrations of acetone and embedded in Epon–Araldite (all reagents were from Electron Microscopy Sciences, Hatfield, PA, USA). Ultrathin sections were cut at a thickness of 70 nm using an Ultracut-E ultramicrotome (Reichert-Jung, Heidelberg, Germany), contrasted with lead citrate, and observed using a Philips Morgagni 268 D electron microscope (Fei Company, Eindhoven, NL, USA) equipped with a Mega View II camera (Olympus, Tokyo, Japan) to acquire digital images.

To confirm the detectability of ASCs labelled with VivoTrax and VivoTrax Plus, MRI acquisitions of agarose gel phantom containing different amounts of labelled cells were performed. Both NPs types were added to ASCs at concentrations of 200, 50, and 6.2 µg Fe/mL. After 24 h and 48 h of incubation, the culture medium was aspirated, and the cells were washed twice with 1X PBS and counted. The designated amounts of cells (100, 1 × 10^3^, 5 × 10^3^, 1 × 10^4^, and 5 × 10^4^) were transferred into 1.5 mL Eppendorf tube containing 1 mL of 1X PBS, followed by centrifugation at 14,000 rpm for 5 min at room temperature. The supernatant was removed and the ASC pellet was resuspended in 10 µL of PBS. A gel matrix phantom (1% agarose in distilled water) was designed with a basin obtained with a 0.2 mL mold before the injection of a cell pellet. MRI acquisitions were performed using a FLASH sequence with TR = 1000 ms, TE = 15 ms, flip angle = 15°, FOV = 65 × 60 mm^2^, MTX = 256 × 192, slice thickness = 0.5 mm, N slice = 45, and NEX = 10.

### 2.3. Extraction and Isolation of EVs from Labelled-ASC Cells

EVs were isolated from the culture medium containing 10^7^ ASC. To isolate labelled EVs, cells were cultured to confluence and incubated for 24 h with 200 µg Fe/mL VivoTrax and VivoTrax Plus, followed by 48 h of FBS deprivation [24]. Cell culture supernatants were collected, and the PureExo^®^ Exosome isolation kit (101 Bio, Palo Alto, CA, USA) was used for EVs isolation, following the manufacturer’s protocol. The protein concentration of EVs was measured by the Bicinchoninic Acid Protein Assay (BCA method) according to the manufacturer’s protocol (Pierce™ BCA™ Protein Assay, Thermo Scientific™, Milan, Italy).

The size and concentration of labelled EVs were measured by NTA using a NanoSight NS300 (Malvern Panalytical, Malvern, UK) equipped with a 488 nm laser and a syringe pump system with a pump speed of 20 µL/s. NTA 3.4 software (Malvern Panalytical) was used to acquire and analyze the sample videos.

TEM (Philips Morgagni 268 D TEM, Fei Company-Philips, Eindhoven, The Netherlands) operated at 80 kV with a Megaview G3 camera (EMSIS Gmbh, Munster, Germany) was used for digital image acquisition of the labelled-EVs deposition on a Formvar/Carbon 300 Mesh Copper (Electron Microscopy Sciences, Hatfield, PA, USA).

### 2.4. Administration of Labelled-EVs to SOD1(G93A) Mice, In Vivo Imaging and Ex Vivo Histology

According to our previously developed protocols [14,24], labelled EVs were tested in SOD1(G93A) mice (n = 3). Animals were maintained under controlled environmental conditions according with the principles of “3R”.

The administration of labelled-EVs was performed at 13–14 weeks of life in SOD1(G93A) mice, the time when typical ALS brain lesions are detected in MRI [25]. Labelled-EVs were administered intranasally through three administrations (one per hour) at a concentration of 20 µg of EVs proteins resuspended in 20 µL PBS. MRI acquisitions were performed prior to administration and 3 and 6 h after administration.

MR images were acquired using a Bruker Tomograph (Bruker, Karlsruhe, Germany) operating a 7.0 T (Oxford Ltd., Oxford, UK). During the scanning procedure, general anesthesia was induced and maintained with 1–2% isoflurane at 0.8 L/min oxygen flow rate through a nose cone. Respiratory rate was monitored during imaging using a pressure-sensitive pad. High-resolution MRI T2-weighted images were acquired using a Rapid Acquisition with Refocused Echoes (RARE) sequence with the following parameters: TR = 5000 ms; TE = 40 ms; FOV = 2.5 × 2.0 cm^2^; MTX = 256 × 256; slice thickness = 0.5 mm; n° of slice = 40; RARE factor = 16; number of averages = 24.

To provide an objective assessment of MRI signal changes following intranasal administration of VivoTrax-labelled EVs, a semi-quantitative analysis of T2-weighted images was performed at baseline and at 3 and 6 h after administration. Because identical animal positioning could not be reproduced across MRI sessions, whole-brain masks were manually generated on three consecutive coronal slices encompassing the lesioned brainstem region. Mean signal intensity was measured within the segmented brain area and normalized to the signal intensity of the ventricular cerebrospinal fluid to minimize inter-scan variability. Statistical analysis was performed using one-way repeated-measures ANOVA followed by Sidak’s multiple-comparison test.

Mice were sacrificed 6 h after in vivo MRI to confirm the presence of NPs by histological analysis. Brains were dissected and fixed in PFA 4% overnight at 4 °C. All samples were embedded in paraffin and cut on a microtome to collect coronal sections (10 µm thick) from all the brains. Perl’s Prussian blue staining was performed to evaluate the presence of the NPs. The sections were incubated with Prussian blue solution (5% hydrochloric acid and 5% potassium ferrocyanide) for 30 min and counterstained with hematoxylin and eosin. The sections were dehydrated using increasing grades of ethanol, cleared in xylene, and cover slipped using Entellan (Merck, Darmstadt, Germany). Images of the sections were acquired using an Olympus APXVIEW APX100 optical microscope (Olympus Italia S.r.l., Segrate, MI, Italy) and examined using the brain mouse atlas to confirm that the area detected by histological analysis overlapped with that detected by MRI.

### 2.5. Statistical Analysis

All experiments were repeated at least three times in triplicate unless otherwise indicated. Data are reported as mean ± standard error of the mean (SEM). Statistical analysis of the data for comparison between two distributions was performed using an unpaired two-tailed *t*-test (GraphPad Prism 8, San Diego, CA, USA). For experiments with more than two conditions, data were tested using one-way or two-way analysis of variance (ANOVA) test (GraphPad Prism). Statistical significance was set at *p* < 0.05.

## 3. Results

### 3.1. Physicochemical Characterization and Imaging Sensitivity

DLS measurements were conducted to assess the hydrodynamic size of VivoTrax and VivoTrax Plus in distilled water (dH2O), PBS, and HEPES (Table 1). The average hydrodynamic size of VivoTrax in water (62.91 ± 0.68 nm) and PBS (70.52 ± 1.51 nm) is comparable to the values reported in the manufacturer’s datasheet. VivoTrax Plus showed a consistent size in water (69.44 ± 0.19 nm) with an increase in PBS (83.93 ± 0.62 nm). Both VivoTrax and VivoTrax Plus exhibited increased size in HEPES (103.32 ± 2.38 nm and 150.1 ± 2.39 nm, respectively). The zeta potential measured in distilled water amounted to −15.7 ± 0.23 mV for VivoTrax and −20.8 ± 4.03 mV for VivoTrax Plus.

To test the stability of the NPs over time, the measurements were repeated after seven days. The average size measured was comparable to that on day 1, except for VivoTrax Plus (116.97 ± 0.23 nm). This increase in size was due to the presence of two peaks, which correlated with the presence of two populations (Supplementary Figure S1). The corresponding PDI values provide additional information on the colloidal stability among the investigated media (Supplementary Table S1). VivoTrax Plus showed a substantially increase in PBS at day 7 (0.556 ± 0.018), indicating a progressive broadening of the particle size distribution and reduced colloidal homogeneity in this medium.

**Figure 1:**
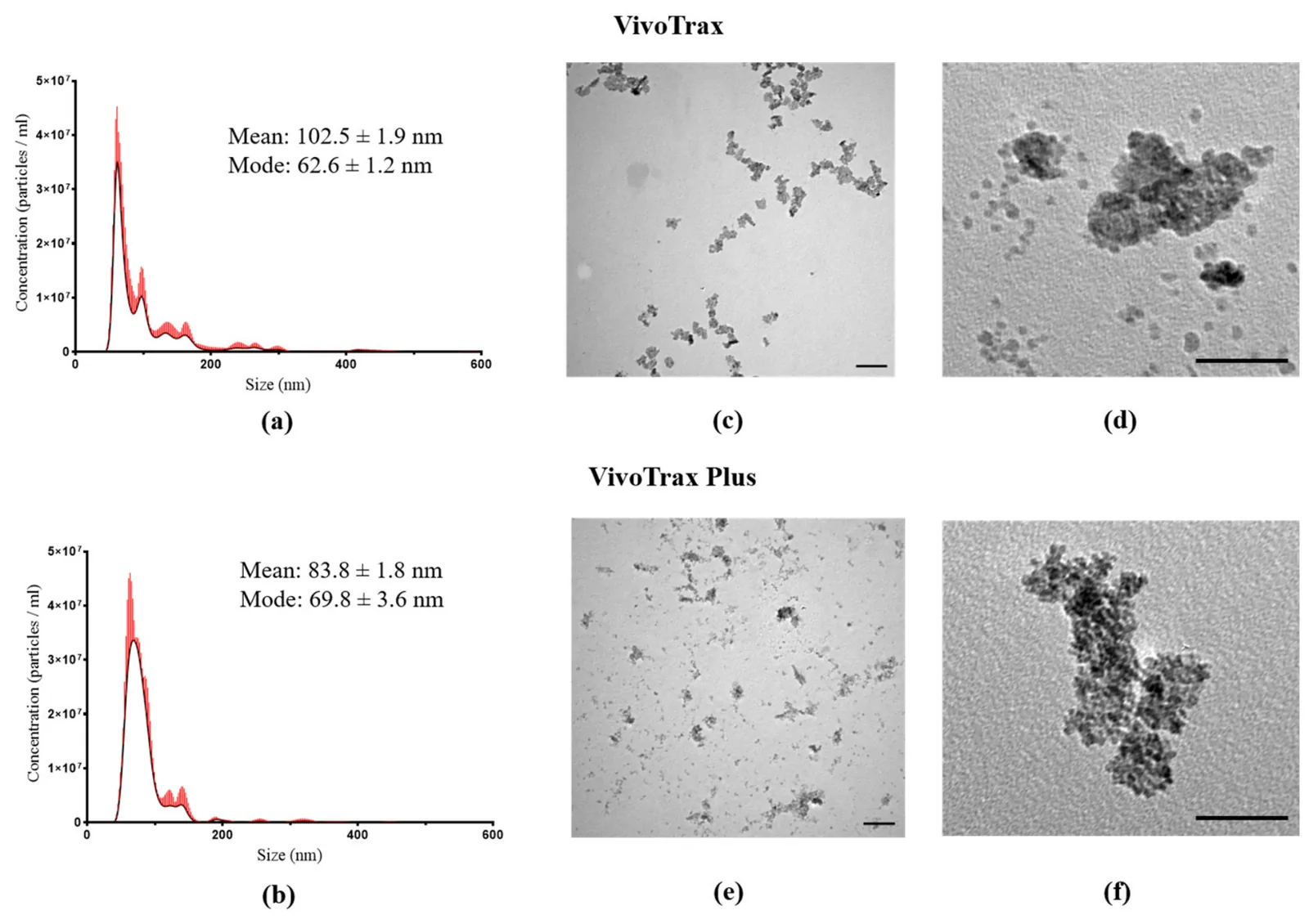
Physochemical Characterization of Nanoparticles. (**a**,**b**) Nanoparticle Tracking Analysis (NTA) on VivoTrax and VivoTrax Plus. (**c**–**f**) Representative transmission electron microscopy (TEM) images acquired at low (44,000×) showing the overall particle distribution ((**c**,**e**), Scale bars: 100 nm). The high magnification (140,000×) of VivoTrax and VivoTrax Plus showing the morphology of individual with the electron-dense iron oxide core and the surrounding coating ((**d**,**f**), Scale bars: 50 nm).

The size distribution of NPs was assessed using NTA measurements after 1:1000 dilution in water (Figure 1a,b). The analysis revealed monodisperse size distribution for both nanoparticle formulations, characterized by distinct primary peaks and the absence of substantial aggregation. The mode size of VivoTrax measured by NTA was 61.5 nm, with a narrow distribution and no significant aggregation. Similarly, VivoTrax Plus NPs exhibited mode size of 68 nm, slightly larger than that of VivoTrax.

TEM analysis was performed at both low and high magnification to evaluate the overall morphology of the nanoparticle preparations and the structural features of individual nanoparticles. Low-magnification images provided a representative overview of the nanoparticle distribution within the samples, revealing well-dispersed nanoparticle populations with minimal aggregation (Figure 1c,e). High-magnification TEM images were acquired to characterize the morphology of individual nanoparticles, revealing the electron-dense iron oxide core and the surrounding coating (Figure 1d,f). Each nanoparticle appeared roughly spherical with an average darker core diameter of approximately 4.5 nm. Additionally, a lighter “halo” surrounding some particles reveals the presence of a stabilizing coating or shell.

**Figure 2:**
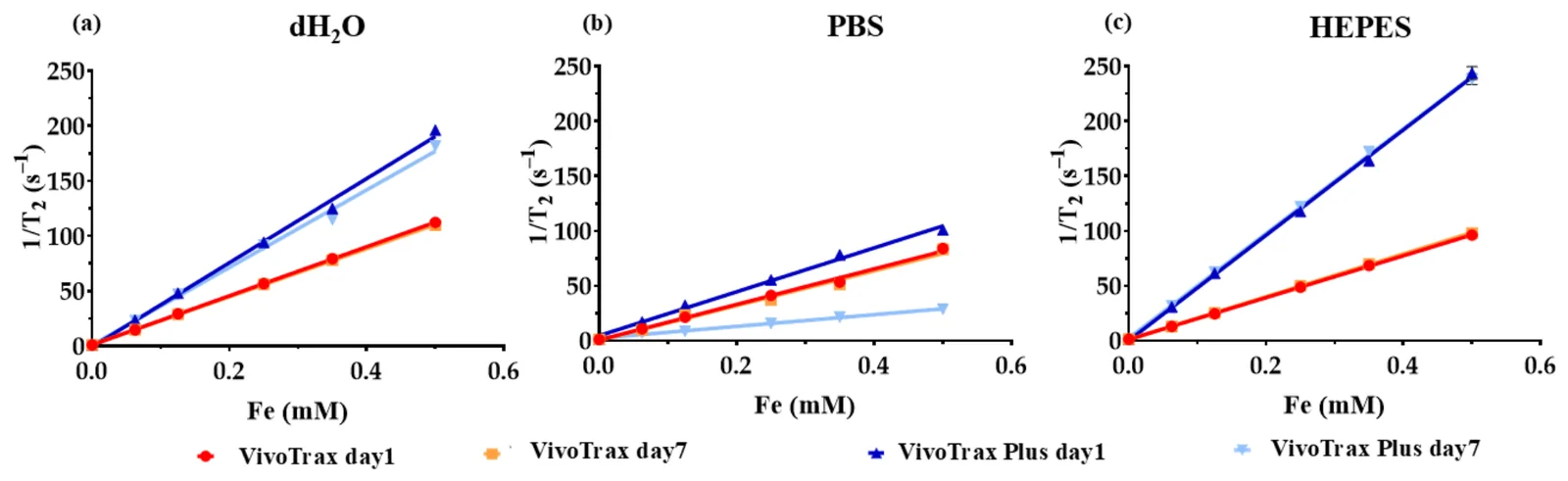
Transverse relaxation rates (1/T2) of VivoTrax and VivoTrax Plus NPs versus Fe concentration in different media: (**a**) dH_2_O, (**b**) PBS, and (**c**) HEPES, at two time points (day 1 and day 7). The data highlight the temporal stability of the relaxometric properties in different environments. The legend, indicating the nanoparticle type and time points, is positioned below the graphs.

The transverse relaxation rates (1/T2) of both NPs as a function of Fe concentration (mM) measured in different media (dH_2_O (A), PBS (B), and HEPES (C)) are shown in Figure 2, while the relaxivity values r2 are listed in Table 2. To assess temporal stability, measurements were repeated seven days after the initial acquisition.

VivoTrax Plus in dH_2_O exhibited higher r2 values than VivoTrax at both time points, suggesting that VivoTrax Plus may provide an enhanced T2 contrast. Moreover, the r2 values in dH_2_O remained nearly constant from day 1 to day 7 for both NPs, indicating excellent temporal stability in this solvent.

The r2 values of both NPs in PBS were lower than in dH_2_O. On day 1, VivoTrax Plus showed a slightly higher r2 value than VivoTrax did. By day 7, VivoTrax Plus showed a marked decrease in r2 (53.5 ± 2.6 mM^−1^s^−1^) compared to VivoTrax (158.1 ± 8.8 mM^−1^s^−1^), suggesting lower temporal stability for VivoTrax Plus in high-salt medium and potentially making VivoTrax more suitable for biological application in PBS.

In HEPES buffer, both NPs displayed good temporal stability, with r2 values remaining consistent between days 1 and 7. Under these conditions, VivoTrax Plus showed higher r2 values than VivoTrax, mirroring the trend observed for dH2O.

Additional transverse (1/T2) relaxation rate data in other media, including DMEM + FBS 10%, self-prepared CSF (Cerebrospinal Fluid), and HEPES + PBS, are provided in the Supplementary Materials (Supplementary Figure S2 and Table S2). These data show how the media composition influences the relaxation properties of NPs. The r2 values of VivoTrax remained approximately constant in all media considered (except for the aCSF), whereas r2 of VivoTrax Plus showed alterations across the media. Of note, a strong decrease in r2 was displayed by VivoTrax Plus in aCSF. Moreover, the trend of both NPs in HEPES + PBS recapitulated an intermediate behaviour compared to HEPES and PBS alone. The longitudinal relaxation rates (1/T1) and longitudinal relaxivity values (r1) of both NPs are reported in the Supplementary Materials (Supplementary Figure S3 and Table S3).

**Figure 3:**
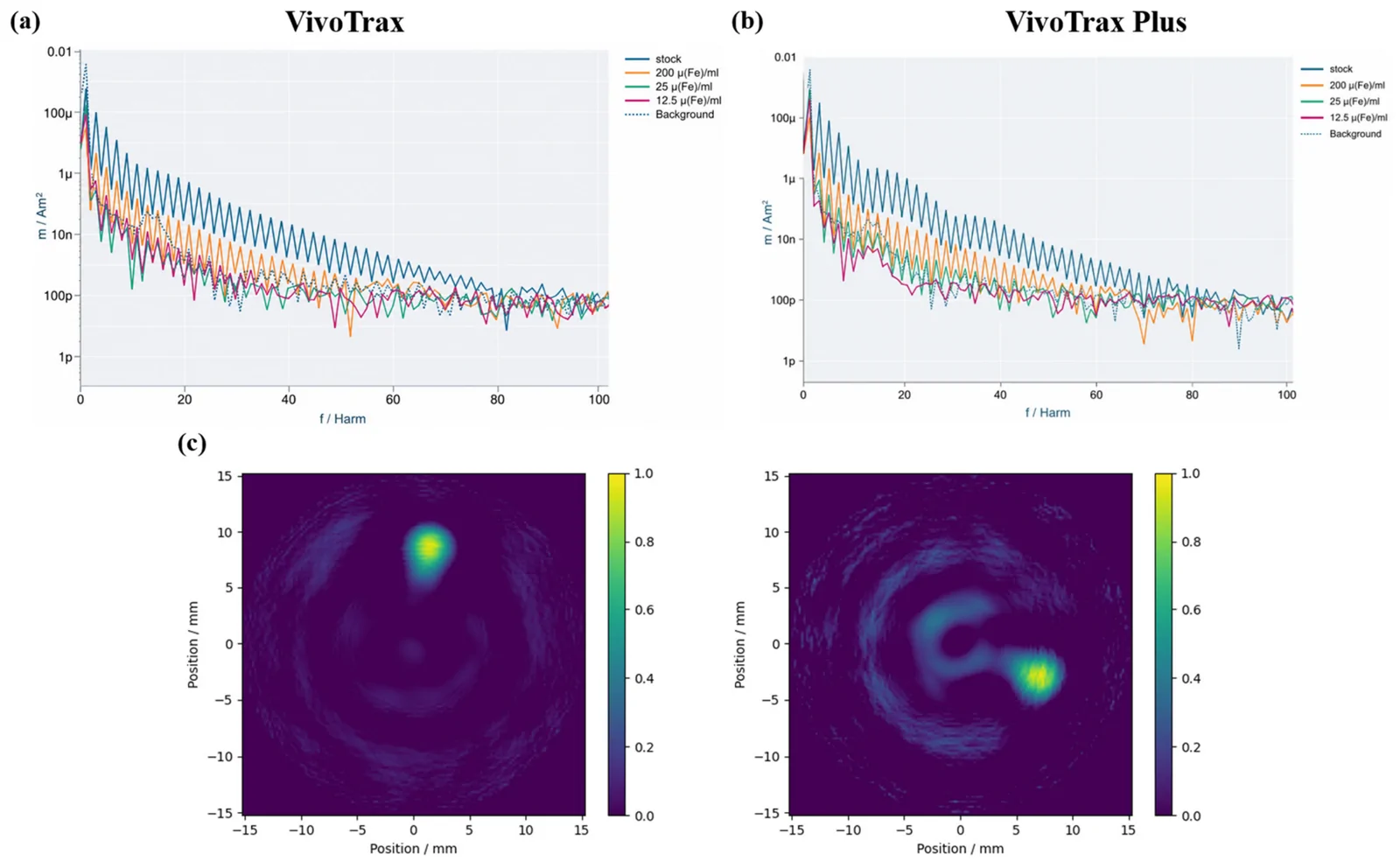
Magnetic Particle Spectroscopy (MPS) results of VivoTrax (**a**) and VivoTraxPlus (**b**). (**c**) Magnetic particle imaging (MPI) results of VivoTraxPlus with 200 µg (Fe)/mL (**left**) and 50 µg (Fe)/mL (**right**).

Magnetic particle spectroscopy (MPS) data, reported in Figure 3a,b, show that the signal decreases with decreasing iron concentration. The signal was reliably detected down to an iron concentration of 25 µg/mL, whereas at lower concentrations it became indistinguishable from the background noise of the system. The performances of both tracers were very similar, with VivoTrax Plus consistently generated higher harmonic amplitudes than VivoTrax, indicating improved MPI performance. In the VivoTrax Plus stock solution, a wave-like pattern was observed, indicating particle–particle interactions.

The results of magnetic particle imaging (MPI) (Figure 3c) show the reconstruction of one slice of the phantom for each dilution of VivoTrax Plus. Both reconstructions show a circular shape corresponding to the cross section of the glass capillary. The sample containing 200 µg(Fe)/mL (left) could be reconstructed with less background noise than the sample containing 50 µg(Fe)/mL, as expected. It should be noted that the positions of the samples did not coincide because measurements were taken individually, and the position of the glass capillary was not deterministic.

### 3.2. Stability in Culture Medium and Cytotoxic Effect on ASCs

**Figure 4:**
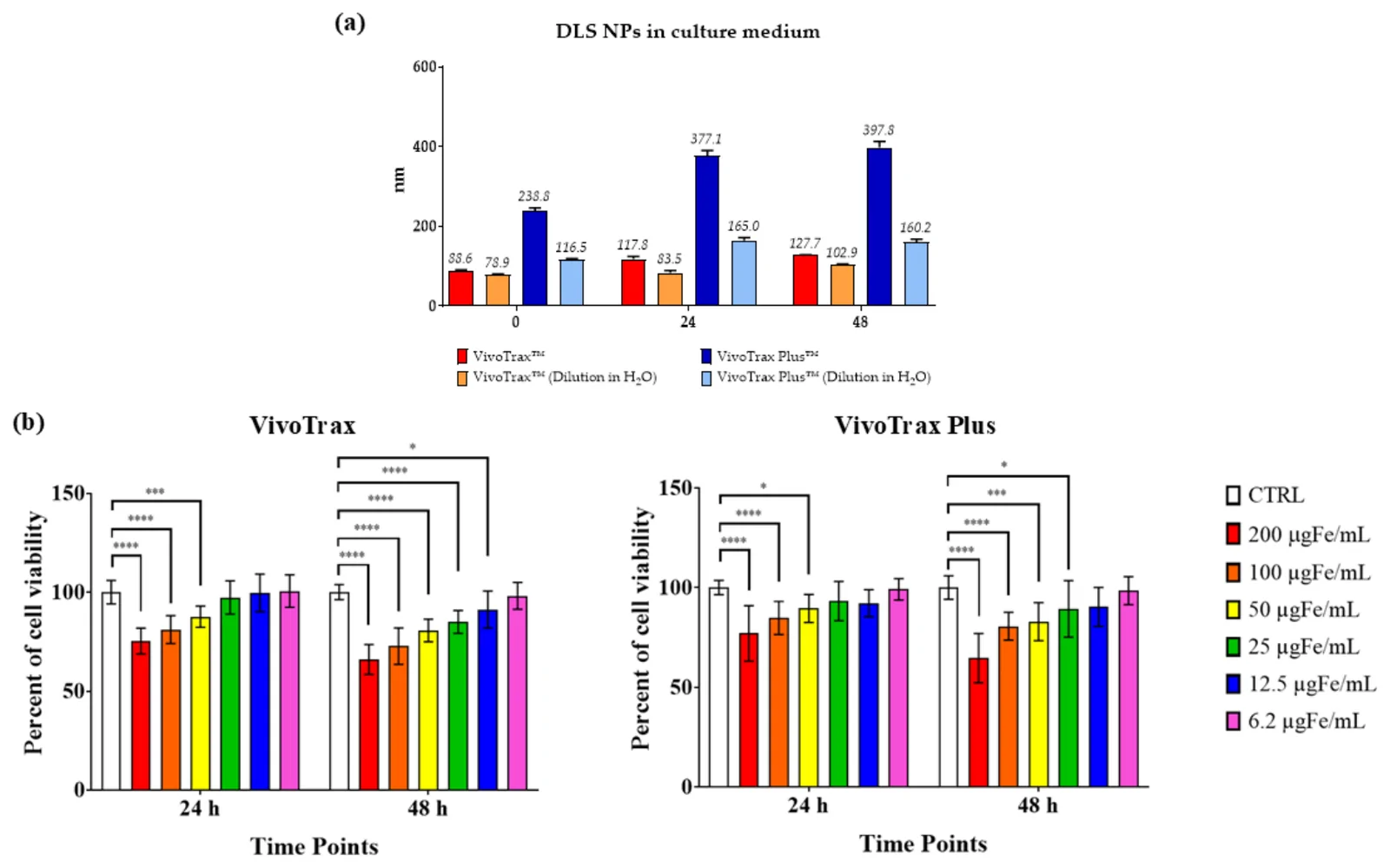
DLS of NPs and MTT assay viability of labelled ASCs. (**a**) DLS of VivoTrax and VivoTrax Plus in culture medium and their diluted version in water (1:1000) at three time points: 0 h, 24 h, and 48 h. (**b**) Effects of VivoTrax and VivoTrax Plus on cell viability at two time points (24 h and 48 h) and different concentrations (200, 100, 50, 25, 12.5, and 6.2 µg Fe/mL). The bar graphs represent the percentage of cell viability at 24 h and 48 h. Viability generally decreases in a time- and dose-dependent manner, with higher concentrations (200, 100, and 50 µg Fe/mL) showing significant reductions in cell viability compared to the control (CTRL). This trend is particularly evident after 48 h. Error bars represent SEM. n = 13. **** *p* < 0.0001; *** *p* < 0.001, * *p* < 0.05.

The hydrodynamic sizes of VivoTrax and VivoTrax Plus were measured by DLS after 0, 24, and 48 h in the culture medium (Figure 4a). VivoTrax Plus presented a significantly larger initial size, compared to water or plain buffer, that further increased from 238 nm to 397 nm over the 48 h time interval. In comparison, VivoTrax showed a moderate increase in size over time, from 88 nm to 127 nm with initial value comparable to that measured in water or plain buffer. These results suggest aggregation phenomena or interactions with the protein component of FBS for VivoTrax Plus.

MTT assay was used to evaluate the cytotoxicity of both NPs formulations, followed by incubation with ASC for 24 and 48 h. A dose-dependent decrease in cell viability was observed (Figure 4b), with statistically significant cytotoxic effects at higher concentrations (200, 100, and 50 µg Fe/mL) after 24 h for both formulations. Prolonged incubation for 48 h resulted in statistically significant cytotoxicity at a concentration of 25 µg Fe/mL for both NPs, indicating a time-dependent effect on ASC viability. Although cell viability decreased to approximately 75% at the highest concentration tested, this labelling condition was selected because it provided sufficient nanoparticle uptake while preserving an adequate number of viable cells for subsequent EV production and imaging studies.

### 3.3. Internalization of Nanoparticles by ASCs

**Figure 5:**
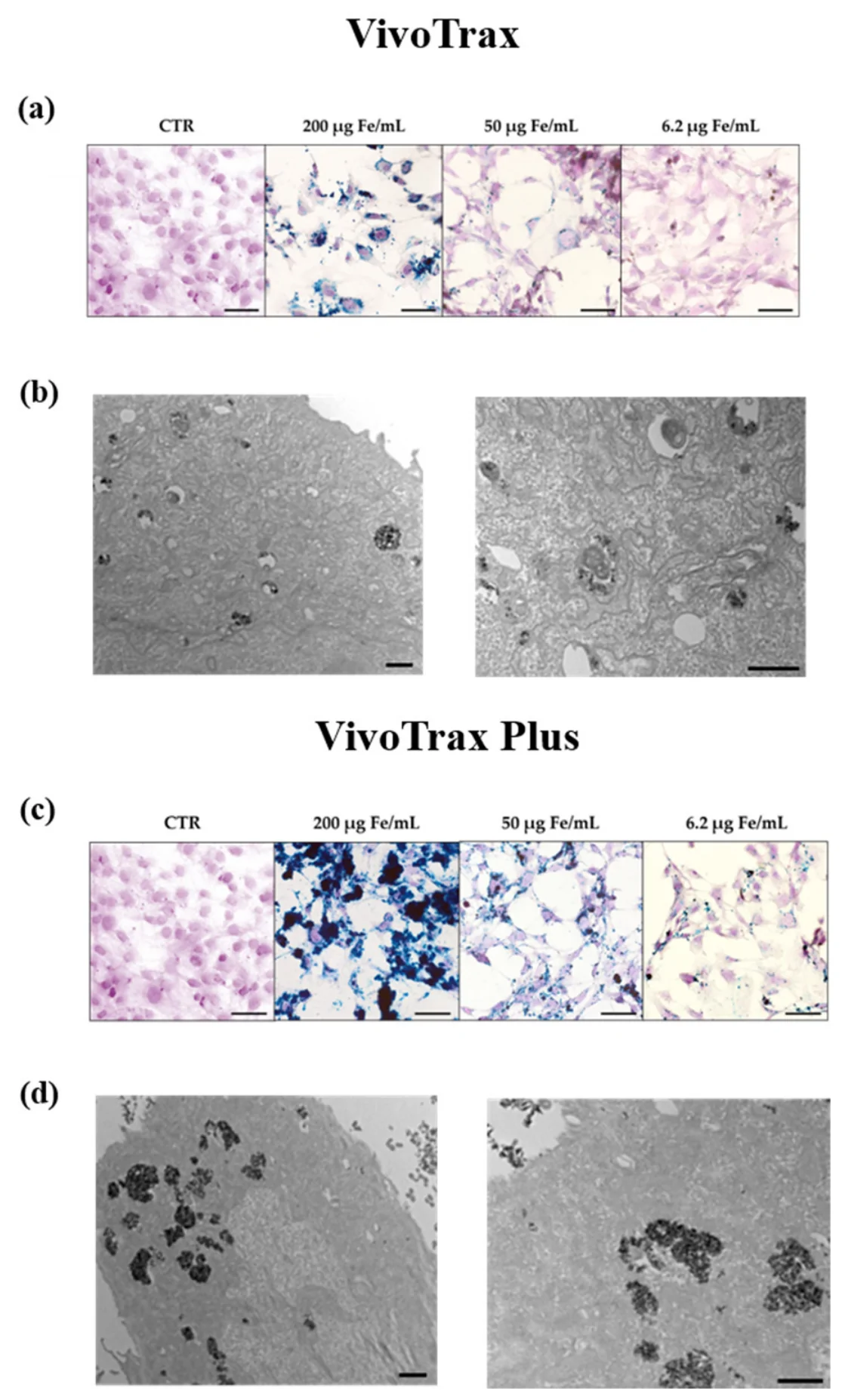
(**a**,**c**) show the histological analysis of labelled ASCs. Prussian Blue staining of ASCs labelled with VivoTrax and VivoTrax Plus at three concentrations (200, 50, and 6.25 µg Fe/mL) Scale bar: 100 µm. (**b**,**d**) Representative TEM images showing the internalization of VivoTrax and VivoTrax Plus by cells. Electron-dense NPs are visible within membrane-bound vesicles, indicating endocytotic uptake. The images show two magnifications to appreciate an overview (7100× magnification), and more detailed images (11,000× magnification) (scale bar: 500 nm).

Prussian Blue staining and TEM analysis were performed to confirm NP internalization by ASCs. Figure 5a,c shows Prussian Blue staining of ASCs labelled with VivoTrax (upper row) and VivoTrax Plus (lower row) at different iron concentrations (200, 50, and 6.2 µg Fe/mL) after 24 h of incubation. Control (CTRL) cells exhibited uniform cytoplasmic staining without detectable blue deposits, confirming the specificity of staining for iron associated with internalized or membrane-bound NPs.

At the highest iron concentration (200 µg Fe/mL), VivoTrax Plus-labelled cells displayed dense, compact, and irregular blue deposits, indicative of NPs aggregation. In contrast, VivoTrax labelling resulted in smaller and more homogeneously distributed blue spots, both within the cytoplasm and along the cell membrane.

At lower concentrations (50 and 6.25 µg Fe/mL), both formulations showed a clear concentration-dependent decrease in the Prussian Blue staining intensity. VivoTrax maintained a relatively homogeneous intracellular distribution, whereas VivoTrax Plus exhibited a less aggregated pattern than the highest concentration, although residual clustering was evident at 50 µg Fe/mL.

TEM images were used to confirm NPs internalization (Figure 5b–d). ASCs incubated for 24 h with VivoTrax (Figure 5b) and VivoTrax Plus (Figure 5d) at 200 µg Fe/mL exhibited electron-dense structures that were localized exclusively within the cytoplasm. The NPs were consistently enclosed within the membrane-bound vesicles, supporting an endocytic uptake mechanism. Once internalized, NPs aggregated into clusters within the vesicular compartments. In several instances, these vesicles displayed morphological features consistent with endo-lysosomal compartments, including heterogeneous and partially electron-lucent content mixed with dense NPs clusters, suggestive of auto-lysosome-like structures, which were particularly evident in VivoTrax Plus-treated cells (Figure 5d). These observations are consistent with intracellular trafficking and processing of NPs along the endocytic-lysosomal pathway. Differences in NP size, clustering, and intracellular localization suggested distinct stages of internalization and vesicular maturation. Notably, no NPs were observed in the cytosol or within the nucleus, indicating an effective vesicular confinement following cellular uptake.

**Figure 6:**
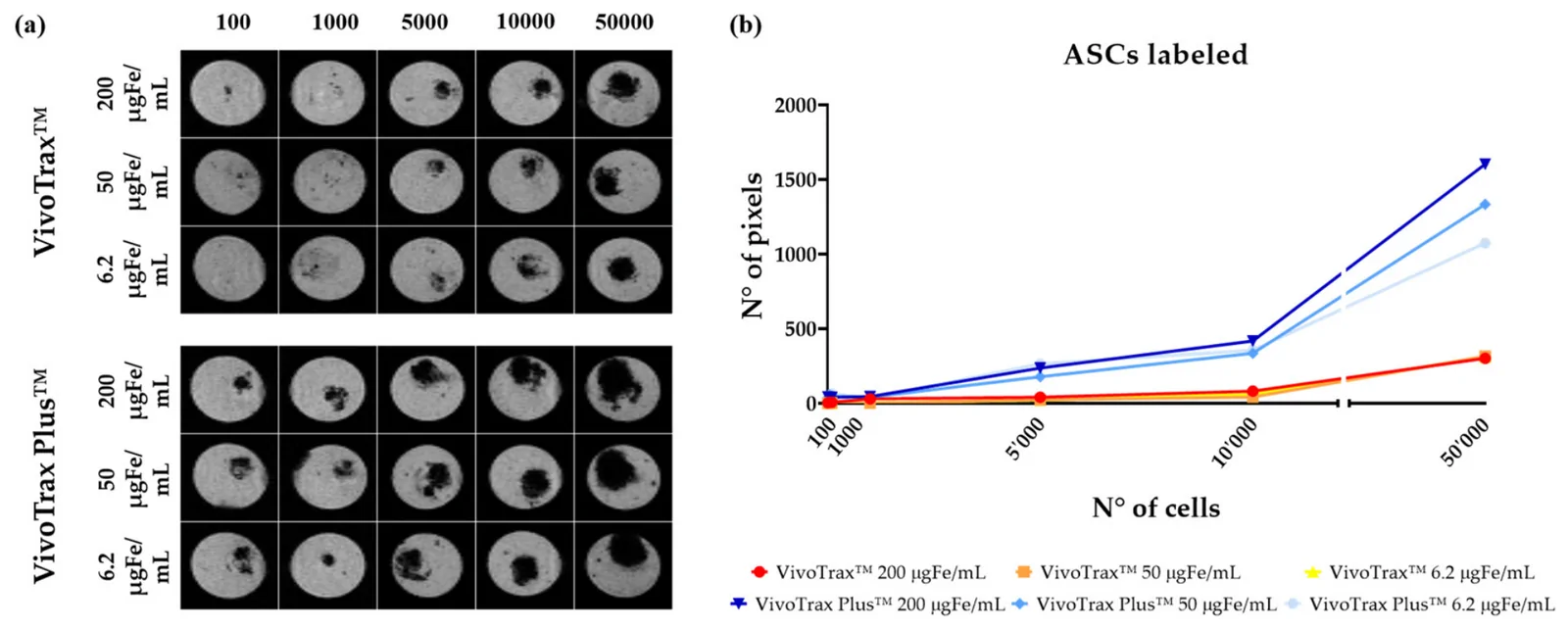
Magnetic resonance analysis (MRI) analysis of labelled-ASC cells. (**a**) Representative MRI images ac-quired using FLASH sequence. Each row corresponds to a different Fe concentration of the NPs, while each column represents the total number of labelled cells. The upper panels display results for VivoTrax, whereas the lower panels show VivoTrax Plus. (**b**) Hypointense regions indicate the presence of labelled ASCs. Quantitative assessment of hypointense regions, represented as the total number of dark pixels across slices.

To assess the MRI detection sensitivity, contrast efficacy, and detection limit, a series of dilutions of cells (ranging from 5 × 10^4^ to 1 × 10^2^) labelled with different Fe concentrations (200, 50, 6.2 µgFe/mL) was performed. Figure 6a shows representative gradient echo (FLASH) MRI images of cell pellets labelled with VivoTrax (top panel) and VivoTrax Plus (bottom panel). Hypointense (dark) regions in the images corresponds to iron, producing magnetic field inhomogeneities that extend beyond the physical boundaries of labelled cells, a phenomenon commonly referred to as “blooming effect.” The spatial extent of these hypointense regions increased with the number of cells, with more cells producing a larger hypointense area and wider signal distortion. Figure 6b provides quantitative analysis by summing the number of dark pixels across all the image slices. Both qualitatively and quantitatively, VivoTrax Plus demonstrated superior performance, producing a higher effect across all tested concentrations. Notably, at the lowest cell number (100), VivoTrax Plus generated clearly detectable hypointense signals at all concentrations, whereas VivoTrax showed a slight signal, also at the highest Fe concentration used for labelling.

### 3.4. Isolation of Labelled EVs from ASCs and Proof-of-Concept of Homing in Brain of SOD1(G93A) Mice

**Figure 7:**
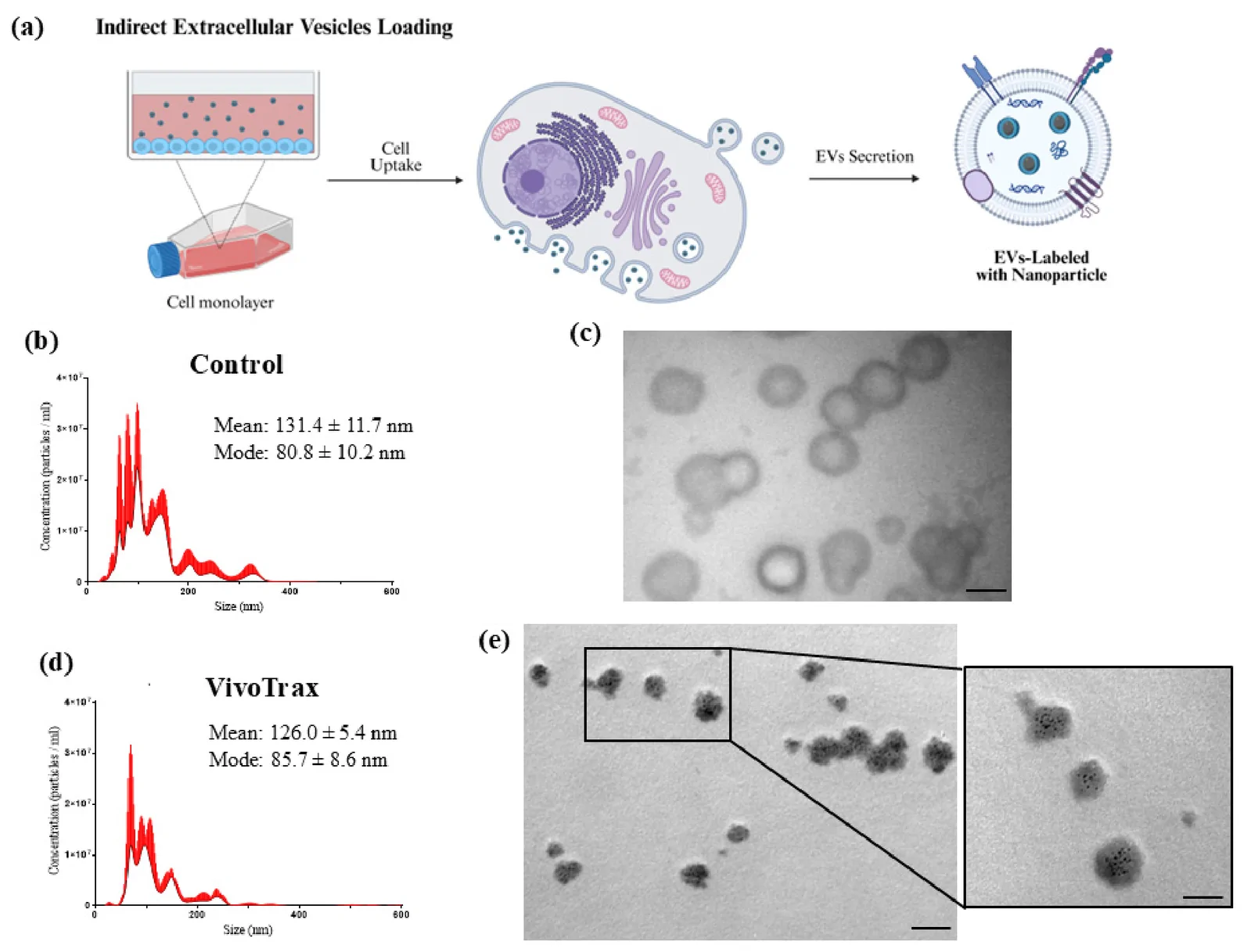
(**a**) Schematic representation of the labelled-EVs production. (**b**,**d**) show the Nanoparticle Tracking Analysis (NTA) on unlabeled EVs and EVs labelled with VivoTrax. Representative transmission electron microscopy (TEM) images showing the control EVs (**c**) and VivoTrax labelled EVs (**e**). Electron-dense NPs were visible within membrane bound vesicles. Two different magnifications to appreciate an overview (56,000× magnification, scale bar: 100 nm) and detailed images (140,000× magnification, scale bar: 100 nm).

A schematic overview of the experimental workflow employed in our laboratory for isolation and extraction of EVs (exosomes fraction 30–150 nm) from previously labelled ASCs is shown in Figure 7a. In previous studies, we have demonstrated that this method [14,26] successfully produced labelled EVs.

NTA measurements were used to check the distribution of EVs isolated from untreated (Figure 7b) and ASCs treated with VivoTrax (Figure 7d). TEM analysis confirmed that the phospholipid bilayer of EVs appeared as a regular, low-density spherical material, suggesting a membrane-like component (Figure 7c,e). Untreated control EVs showed a double phospholipid layer surrounding a less-dense lumen (Figure 7c). In contrast, the internal lumen of VivoTrax labelled-EVs was filled with NPs spots, showing hyperintense contrast (Figure 7e). Notably, the same procedure performed with VivoTrax Plus did not produce labelled-EVs within the isolated 30–150 nm EV fraction as detected by TEM analysis. Importantly, NTA confirmed the successful isolation of EVs with comparable size distribution and particle concentration (Supplementary Figure S4).

**Figure 8:**
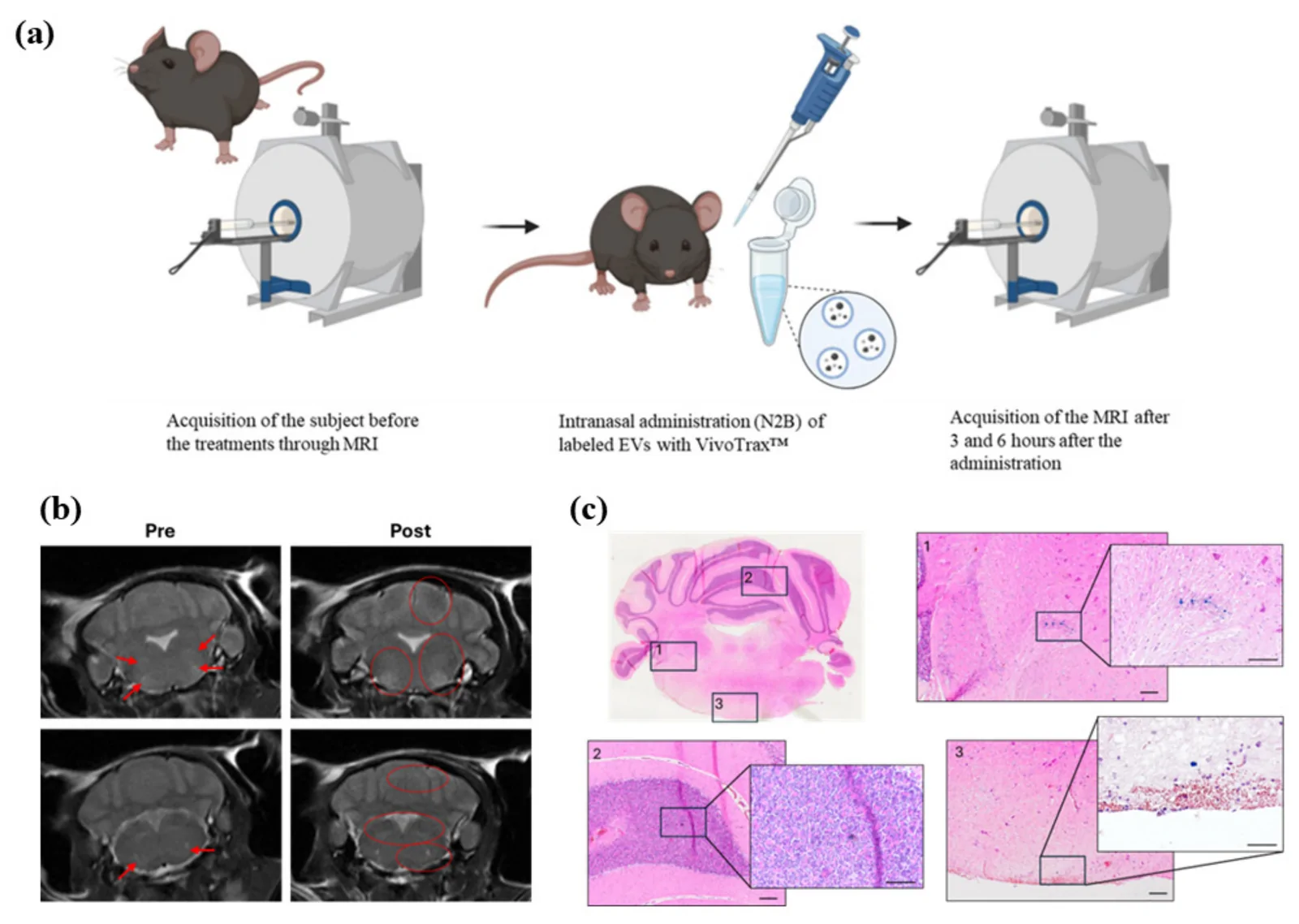
In vivo MR images and histological analysis of SOD1(G93A) mice brain. (**a**) Schematic protocol of MR acquisition. (**b**) This panel showed representative in vivo MR images acquired prior (preMRI), to identify typical lesioned area of the SOD1(G93A) mice (see red arrow), and 6 h post intranasal administration of EVs labeled with VivoTrax. The red circle marked the area in which a hypointense spots is present, attributable to the presence of NPs. (**c**) Prussian Blue staining of the comparable region of the coronal mouse brain map shows blue spots to the same area revealed by MRI, confirming the presence of NPs. The images show the coronal mouse brain slice and two different magnifications to appreciate an overview (10× magnification, scale bar: 100 µm) and more detailed images (20× magnification, scale bar: 100 µm).

To demonstrate the ability of ASC-EVs to reach the CNS, EVs labelled with VivoTrax were administered intranasally to SOD1(G93A) mice at 13–14 weeks of age when typical ALS brain lesions were detected by MRI [25]. A schematic workflow of the administration protocol is shown in Figure 8a.

T2-weighted images of the brain were acquired to identify lesioned areas localized in brainstem regions, corresponding to the trigeminal and facial motor nuclei. Hyperintense lesions were clearly detectable in the images acquired prior to EV administration (red arrows in Figure 8b). Representative MR images acquired 3 and 6 h after intranasal administration of labelled EVs showed no evident signal changes at 3 h, whereas hypointense regions became detectable within the lesion area after 6 h (see the areas enclosed by circular regions in Figure 8b and Supplementary Figure S5A). To further support the qualitative MRI observations, a semi-quantitative analysis of normalized signal intensity demonstrated a progressive decrease over time, reaching an approximately 8% signal reduction 6 h after EV administration (Supplementary Figure S5B). Although the trend was consistent among animals, no statistically significant differences were detected, most likely because of the limited sample size (n = 3). Histological analysis performed by Prussian Blue staining confirmed the presence of iron in the same injured area of the mouse brain, supporting the accumulation of EVs to the lesioned sites in ALS (Figure 8c). Representative MRI and histological images obtained from control SOD1 (G93A) mice receiving unlabeled EVs were also included (Supplementary Figure S5C,D). No focal hypointense MRI signal or Prussian Blue-positive staining was detected in the corresponding brain regions, supporting the specificity of the MRI findings and confirming the absence of non-specific iron staining in control animals.

## 4. Discussion

NP-based contrast agents play a crucial role in non-invasive imaging, both in preclinical and clinical research [13]. Among these, SPIONs have been widely used as contrast agents for MRI owing to their high sensitivity and biocompatibility. Physicochemical differences among SPION formulations can have a significant effect on their performance in imaging as well as in interactions with the biological environment. In this study, we compared two commercially available SPIONs, in terms of their physicochemical characteristics, cytotoxicity, and imaging performance, to explore novel contrast agents for MRI and MPI. VivoTrax and VivoTrax Plus are synthesized by the co-precipitation of iron salts in a reducing environment and have a carboxy dextran coating for colloidal stabilization in physiological fluids [9,10]. Physical and chemical characterization via DLS and NTA revealed that VivoTrax exhibited a hydrodynamic size consistent with the manufacturer’s datasheet, while VivoTrax Plus displayed a modestly larger hydrodynamic diameter. These data confirmed the monodispersity and absence of significant aggregation under aqueous conditions, supporting the stability of the NPs formulations. The evaluation of various dispersion media revealed medium-dependent changes in the NPs size. DLS measurements showed increased sizes in PBS and HEPES compared to water, which can be attributed to various physicochemical properties, including electrostatic interactions, ionic strength, and NPs surface. Overall, both formulations demonstrated good stability over time in all three tested solutions, except for VivoTrax Plus in PBS, which showed an increase of approximately 39% in size on day 7. Generally, ionic strength plays a crucial role, and its increase leads to higher NPs interaction/aggregation, corresponding to a larger hydrodynamic size [27,28]. It has been reported that uncoated NPs tend to flocculate immediately in high ionic strength solutions like PBS [27].

The behaviour of SPIONs in complex biological environments is of particular interest for imaging applications. Our analysis confirmed that both formulations exhibited minimal longitudinal relaxivity (r1), thus preventing their use as contrast agents in T1-weighted MRI (Supplementary Figure S3 and Table S3). Transversal relaxivities of VivoTrax were in good agreement with our previous paper [29]. Compared to Vivotrax, VivoTrax Plus exhibits a substantially higher transversal relaxivity (r2), making it more effective for T2-weighted imaging. Overall, the r2 values for both NPs remained stable over time, showing only modest variation. Specifically, both water and HEPES did not affect the r2 value of both NPs, while in PBS, the r2 value of VivoTrax Plus decreased over time (from 199.9 mM^−1^s^−1^ to 53 mM^−1^s^−1^, ~73.5% decrease). Further analysis in biologically relevant media (DMEM + 10% FBS, CSF, and HEPES + PBS reported in the Supplementary) revealed that VivoTrax exhibited a higher and more stable r2 value over time than VivoTrax Plus. An interesting finding of this study was the marked reduction in the transverse relaxivity of VivoTrax Plus when dispersed in PBS and aCSF, whereas only minor changes were observed in water and HEPES. This behaviour suggests that the magnetic performance of SPIONs is strongly influenced by the physicochemical properties of the surrounding medium. PBS and aCSF reproduce physiological ionic environments and contain relatively high concentrations of mono- and divalent ions, which can modify electrostatic interactions between nanoparticles and reduce their colloidal stability. Additionally, DLS results (Table 1) showed an increased hydrodynamic size, suggesting the occurrence of aggregation/flocculation. Consistent with this interpretation, DLS measurements (Table 1) showed an increase in both hydrodynamic diameter and PDI, particularly for VivoTrax Plus, suggesting the occurrence of aggregation/flocculation under these conditions. These findings emphasize that SPION relaxivity should be evaluated in biologically relevant media, as measurements performed exclusively in water may not accurately predict in vivo MRI performance. VivoTrax Plus consistently exhibited higher harmonic amplitudes than VivoTrax across all tested concentrations, indicating improved magnetic performance. This observation is consistent with previous reports showing enhanced MPI sensitivity and resolution for the magnetically fractionated VivoTrax Plus formulation [9].

ASC viability was assessed following internalization of the two NPs formulations to evaluate their cytotoxicity and suitability for cell labelling and tracking applications. Overall, ASC remained viable after incubation with both NPs, even at the highest concentrations tested. Although a statistically significant reduction in cell viability was observed, at 200 µg Fe/mL, this level of viability was considered acceptable for the intended application and was therefore selected as a suitable NP dose to ensure sufficient intracellular labelling for MRI detection. In a previous study conducted by our group using Resovist^®^ under comparable experimental conditions, no significant difference was reported in cell viability between the control and treated cells [26]. Notably, despite the close similarity between VivoTrax and Resovist^®^, the observed differences in ASC viability suggest that subtle variations in NPs formulation or physicochemical properties may influence cellular responses. Prussian Blue staining at comparable Fe concentrations indicated effective NPs uptake by ASCs in both the formulations. However, VivoTrax Plus exhibited pronounced staining, likely associated with a higher degree of NPs aggregation, in agreement with DLS measurements and MRI relaxivity analysis. TEM data were consistent with the Prussian Blue staining results, supporting a higher level of intracellular clustering for VivoTrax Plus than for VivoTrax. NPs were detected within cytoplasmic vesicles and multivesicular bodies (MVBs) indicating that internalization occurred predominantly through endocytic mechanisms. MVBs fuse with the plasma membrane and release their contents including EVs into the extracellular space [30]. Additionally, the coexistence of vesicles containing homogenous electron-dense material and compartments characterized by heterogeneous, partially electron-lucent content suggests progressive vesicular maturation along the endocytic-lysosomal pathway. In line with these observations, Gevaert et al. also reported a higher tendency for VivoTrax Plus to aggregate than VivoTrax [9].

MRI of the ASC-labelled phantoms demonstrated a clear relationship between cell number and signal loss, with blooming artefacts typical of T2*-weighted sequences. VivoTrax Plus exhibited higher sensitivity and detectability than VivoTrax even at low cell counts (100 cells). This may be partially explained by the increased transverse relaxivity, which enhances the magnetic susceptibility effects. Using the experimental protocol applied in this study, labelled EVs were obtained using only VivoTrax. NTA data showed no significant difference in the EVs population extracted from labelled cells compared with the control. In labelled-EVs the mode particle size remained consistent at approximately 86 nm, suggesting that the NPs were effectively incorporated into EVs without significantly altering their dimensions. This result is in line with the expected size of EVs isolated by the PureExo^®^ Exosome Isolation Kit, which selectively collects the fraction of small dimension commonly identified as “exosomes” [23,24,26]. Unfortunately, efficient labelling was not observed with VivoTrax Plus. One possible explanation for these results is the pronounced intracellular accumulation of NPs in ASC, as evidenced by Prussian Blue staining and TEM analysis (Figure 5b,d). This observation suggests that VivoTrax Plus may be preferentially retained within cells or sequestered into distinct intracellular compartments, limiting their incorporation into EVs. We cannot exclude with the present data, the possibility that VivoTrax Plus may be preferentially incorporated into larger EV subpopulations (>200 nm).

To test the homing of EVs labelled with VivoTrax, we performed a pilot study following a consolidated protocol for intranasal delivery of EVs [31]. Intranasal administration is a non-invasive procedure that allows repetitive dispensation and efficient delivery of vesicles to the brain. These characteristics make this type of administration an efficient approach for delivering EVs to the lesioned area involved in ALS neurological disease [14,24]. T2-weighted MRI performed at 3 h and 6 h after administration, showed that at 3 h did not reveal visually appreciable hypointense regions, whereas focal hypointensities became evident after 6 h. Semi-quantitative analysis of normalized signal intensity, indicated a progressive reduction following intranasal administration with an approximately 8% after 6 h. While this trend did not reach statistical significance because of the limited number of animals included in this proof-of-concept study, it was consistently associated with the appearance of focal hypointense regions on MRI and with Prussian Blue-positive staining. Prussian blue staining confirmed the presence of iron in the corresponding lesioned brain areas as detected by MRI. Moreover, the absence of MRI signal alterations and iron staining in control animals receiving unlabeled EVs further supports the specificity of the observed imaging findings. A limitation of the present study is that the administered EV preparations were normalized according to protein content rather than iron content. Although protein normalization is commonly used in EV studies, direct quantification of SPION-associated iron would enable a more accurate correlation between the administered dose and MRI sensitivity. Further experiments are needed to clarify the mechanisms of action of EVs and cell type internalization.

## 5. Conclusions

In conclusion, this study compared two commercially available SPION-based contrast agents, VivoTrax and VivoTrax Plus, and highlighted the influence of physicochemical properties on NPs stability, cellular interactions, and imaging performance. While VivoTrax Plus exhibits higher transverse relaxivity in MRI and signal intensity in MPI, VivoTrax shows greater stability over time in biologically relevant media. These differences resulted in distinct biological outcomes. Although both formulations were efficiently internalized by ASC with acceptable cytotoxicity, only VivoTrax enabled effective and reproducible EVs labelling using an indirect production strategy. Our results indicate that NPs stability and intracellular trafficking are key factors of labelling efficiency, rather than relaxometric performance alone. Notably, indirect labelling approach preserves the size distribution of EVs and exploits endogenous vesicle biogenesis pathways, thereby minimizing structural alterations and enhancing translational relevance. The successful in vivo detection of VivoTrax-labelled EVs in a pilot ALS mouse model further supports its potential as a versatile and translatable platform for theranostic applications.

## Figures and Tables

**Table 1:** DLS and Zeta Potential analysis of nanoparticles at Day 1 and Day 7.

	Zeta Potential (mV ± SDev)	Size on DLS (nm ± SDev)	Zeta Potential (mV ± SDev)	Size on DLS (nm ± SDev)
	Day 1	Day 1	Day 7	Day 7
VivoTrax in dH_2_O	−15.7 ± 0.23	62.91 ± 0.68	−15.9 ± 1.05	60.33 ± 2.73
VivoTrax in PBS		70.52 ± 1.51		74.55 ± 2.32
VivoTrax in HEPES		103.32 ± 2.38		97.26 ± 2.35
VivoTrax Plus in dH_2_O	−20.8 ± 4.03	69.44 ± 0.19	−14.71 ± 0.75	69.90 ± 0.86
VivoTrax Plus in PBS		83.93 ± 0.62		116.97 ± 0.23
VivoTrax Plus in HEPES		150.1 ± 2.3		157.4 ± 1.88

**Table 2:** Summary of r2 ± SD values of the nanoparticles in different media at Day 1 and Day 7.

	VivoTrax r_2_ (mM^−1^s^−1^)		VivoTrax Plus r_2_ (mM^−1^s^−1^)	
	Day 1	Day 7	Day 1	Day 7
dH_2_O	222.9 ± 0.6	218.1 ± 1.0	382.0 ± 11.9	351.9 ± 13.0
PBS	162.0 ± 5.5	158.1 ± 8.8	199.9 ± 7.9	53.5 ± 2.6
HEPES	190.7 ± 1.0	194.8 ± 1.1	479.8 ± 7.6	476.5 ± 5.2

## Data Availability

All raw data related to this publication are available from the corresponding author upon reasonable request.

## Supplementary Materials

The following supporting information can be downloaded at https://www.mdpi.com/article/10.3390/nano16161022/s1, Figure S1: DLS size distribution profiles by intensity of VivoTrax and VivoTrax Plus measured on day 1 and 7. The measurements were performed in various solution (dH_2_O, PBS, HEPES). Each panel displays size distribution curves obtained from three replicate measurements (represented in red, green, and blue). Changes in the hydrodynamic diameter and distribution width over time indicate the influence of solution conditions on nanoparticle stability and aggregation behaviour. The red arrow underlines the presence of two population for the VivoTrax Plus in PBS; Figure S2: Transverse relaxation rates (1/T2) of VivoTrax and VivoTrax Plus nanoparticles versus Fe concentration in different media: (a) DMEM+FBS 10%, (b) self-prepared CSF, and (c) HEPES+PBS buffer, at two time points (day 1 and day 7). The data highlight the temporal stability of the relaxometric properties in different environments. The legend, indicating the nanoparticle type and time points, is positioned below the graphs; Figure S3: Longitudinal relaxation rates (1/T1) of VivoTrax and VivoTrax Plus NPs versus Fe concentration in different media: (a) dH_2_O, (b) PBS, (c) HEPES, (d) DMEM + FBS10%, (e) aCSF, and (f) HEPES+PBS buffer, at two time points (day 1 and day 7). The data highlight the temporal stability of the relaxometric properties in different environments. The legend, indicating the nanoparticle type and time points, is positioned below the graphs; Figure S4: (a) Nanoparticle Tracking Analysis (NTA) showing the representative size distribution of the isolated EV population, consistent with the expected size range of small extracellular vesicles. (b) Representative TEM image (56,000× magnification) of EVs isolated from VivoTrax Plus-treated ASCs. No electron-dense NPs were visible within membrane-bound vesicles, supporting the absence of NPs into the isolated 30–150 nm EV fraction. Scale bar: 100 nm; Figure S5: (a) Representative T2-weighted MR images from an additional SOD1(G93A) mouse showing preMRI (baseline), 3 h and 6 h after intranasal administration of EVs labeled with VivoTrax. The red circle marked the area in which a hypointense spots is present. (b) Semi-quantitative analysis of normalized whole-brain signal intensity in the three treated animals (mean ± SD). Signal intensity was normalized to the ventricular cerebrospinal fluid signal and averaged over three consecutive coronal slices. (c) Representative T2-weighted MR images from a control SOD1(G93A) mouse receiving unlabeled EVs. (d) Representative Prussian Blue staining of the corresponding control brain, showing the absence of iron-positive staining the lesioned area. The images show the coronal mouse brain slice (10× magnification, scale bar: 100 µm). Table S1: Polydispersity index (PDI) values (mean ± SD) of VivoTrax and VivoTrax Plus in different media at Day 1 and Day 7. Table S2: Summary of r2 ± SD values of the nanoparticles of VivoTrax and VivoTrax Plus dispersed in different media at Day 1 and Day 7. Table S3: Summary of *r*_1_ ± SD values of the nanoparticles.

## Abbreviations

**ASCs**, Adipose Stem Cells; **CSF**, cerebrospinal fluid; **CTRL**, control group; **DLS**, dynamic light scattering; **EV**, Extracellular Vesicle; **FBS**, Fetal Bovine Serum; **FLASH**, Fast Low-Angle Signal; **MPS**, Magnetic Particle Spectroscopy; **MPI**, Magnetic Particle Imaging; **MRI**, Magnetic Resonance Imaging; **MTX**, Matrix Size; **MVB**, MultiVesicular Body; **NTA**, Nanoparticle Tracking Analysis; **PBS**, Phosphate-Buffered Saline; **SPION**, Superparamagnetic Iron-Oxide Nanoparticle; **USPIO**, Ultra-Small Superparamagnetic Iron-Oxide Nanoparticle; **TEM**, Transmission Electron Microscopy.
