# Supplementary material for: Sensitivity and Cellular Labelling Performance of Magnetically Fractionated SPIONs for Multimodal MRI/MPI Imaging
Source: Nanomaterials (Basel). 2026 Aug 18;16(16):1022. doi: 10.3390/nano16161022 (PMC13516920; doi:10.3390/nano16161022)
Supplement: Supplementary file 1 [file nanomaterials-16-01022-s001.zip › nanomaterials-4437776-supplementary.pdf]

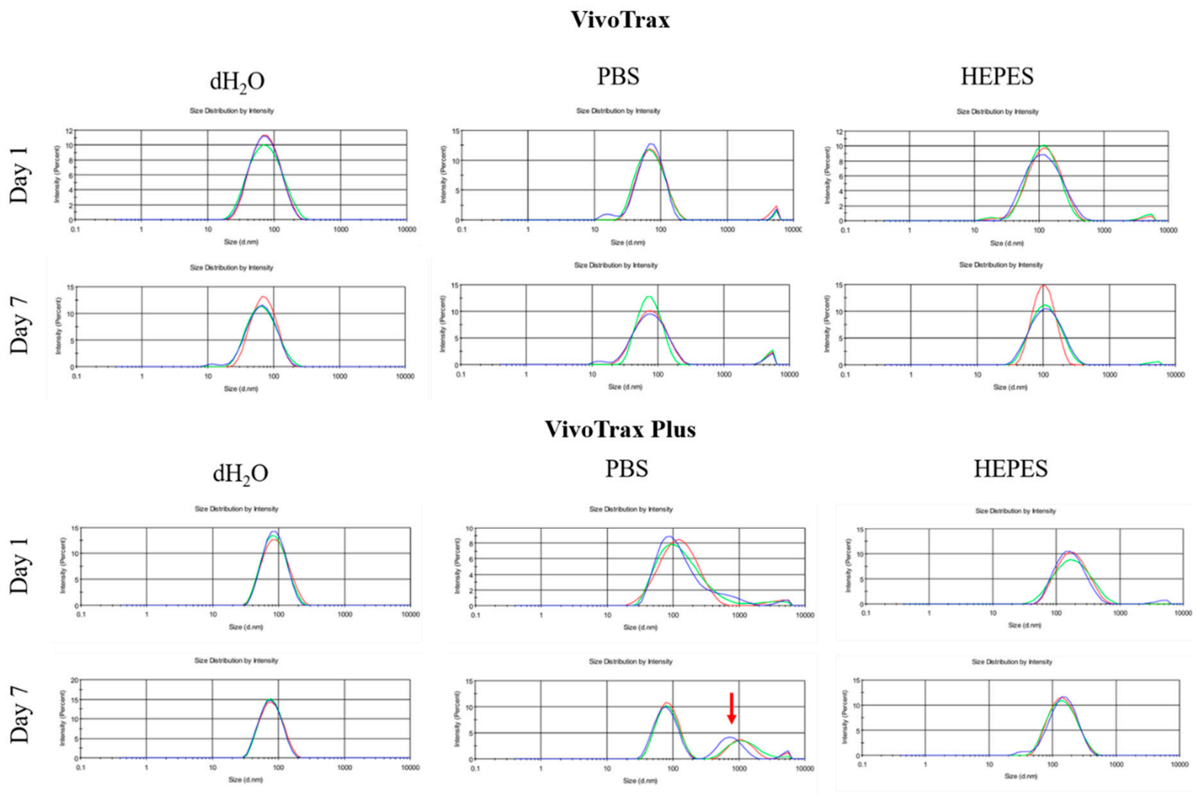

Supplementary Figure S1. DLS size distribution profiles by intensity of VivoTrax and VivoTrax Plus measured on day 1 and 7. The measurements were performed in various solution (dH2O, PBS, HEPES). Each panel displays size distribution curves obtained from three replicate measurements (represented in red, green, and blue). Changes in the hydrodynamic diameter and distribution width over time indicate the influence of solution conditions on nanoparticle stability and aggregation behaviour. The red arrow underlines the presence of two population for the VivoTrax Plus in PBS

Supplementary Table S1. Polydispersity index (PDI) values (mean ± SD) of VivoTrax and VivoTrax Plus in different media at Day 1 and Day 7.

	**Polidispersity Index (PDI) (mean ± SD)**	
	**Day 1**	**Day 7**
VivoTrax in dH2O	0.220 ± 0.014	0.192 ± 0.014
VivoTrax in PBS	0.322 ± 0.021	0.344 ± 0.032
VivoTrax in HEPES	0.264 ± 0.005	0.268 ± 0.008
VivoTrax Plus in dH2O	0.188 ± 0.011	0.158 ± 0.009
VivoTrax Plus in PBS	0.384 ± 0.012	0.556 ± 0.018
VivoTrax Plus in HEPES	0.242 ± 0.016	0.221 ± 0.011

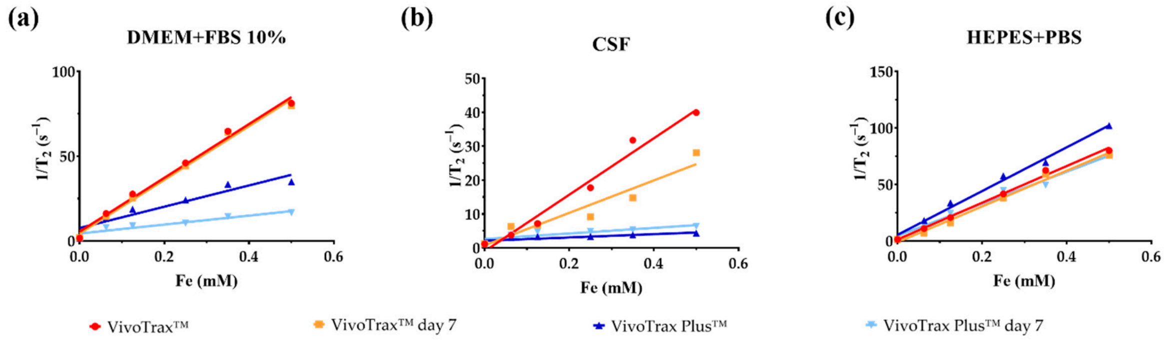

Supplementary Figure S2. Transverse relaxation rates (1/T_2) of VivoTrax and VivoTrax Plus nanoparticles versus Fe concentration in different media: (a) DMEM+FBS 10%, (b) self-prepared CSF, and (c) HEPES+PBS buffer, at two time points (day 1 and day 7). The data highlight the temporal stability of the relaxometric properties in different environments. The legend, indicating the nanoparticle type and time points, is positioned below the graphs.

Supplementary Table S2. Summary of r_2 ± SD values of the nanoparticles of VivoTrax and VivoTrax Plus dispersed in different media at Day 1 and Day 7.

	**VivoTrax r_2 (mM^{-1} s^{-1})**		**VivoTrax Plus r_2 (mM^{-1} s^{-1})**	
	**Day 1**	**Day 7**	**Day 1**	**Day 7**
DMEM+FBS10%	158.7 ± 8.2	158.4 ± 7.4	62.7 ± 10.8	26.4 ± 4.2
CSF	83.3 ± 6.2	47.9 ± 7.3	4.9 ± 1.5	8.2 ± 2.3
HEPES+PBS	162.6 ± 6.1	158.2 ± 7.9	193.8 ± 9.3	141.8 ± 9.1

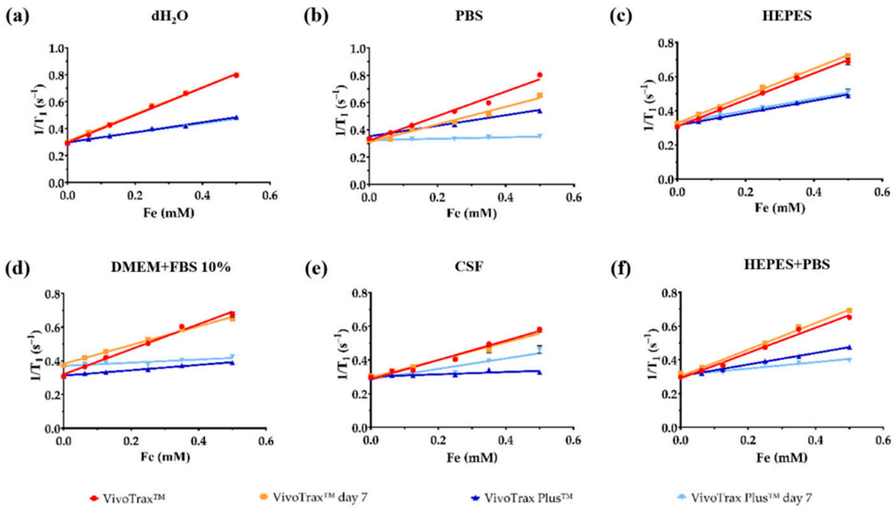

Supplementary Figure S3. Longitudinal relaxation rates (1/T_1) of VivoTrax and VivoTrax Plus NPs versus Fe concentration in different media: (a) dH_2O, (b) PBS, (c) HEPES, (d) DMEM+FBS10%, (e) aCSF, and (f) HEPES+PBS buffer, at two time points (day 1 and day 7). The data highlight the temporal stability of the relaxometric properties in different environments. The legend, indicating the nanoparticle type and time points, is positioned below the graphs.

Supplementary Table S3. Summary of $r_1 \pm$ SD values of the nanoparticles.

	**VivoTrax** $\mathbf{r_1}$ **(mM** $^{-1}$ **s** $^{-1}$**)**		**VivoTrax Plus** $\mathbf{r_1}$ **(mM** $^{-1}$ **s** $^{-1}$**)**	
	**Day 1**	**Day 7**	**Day 1**	**Day 7**
dH_2O	1.0 ± 0.02	1.0 ± 0.02	0.4 ± 0.01	0.3 ± 0.01
PBS	0.9 ± 0.06	0.6 ± 0.05	0.4 ± 0.03	0.1 ± 0.01
HEPES	0.8 ± 0.02	0.8 ± 0.1	0.4 ± 0.02	0.4 ± 0.02
DMEM+FBS10%	0.7 ± 0.04	0.6 ± 0.02	0.2 ± 0.01	0.1 ± 0.02
CSF	0.6 ± 0.05	0.5 ± 0.04	0.1 ± 0.02	0.3 ± 0.06
HEPES+PBS	0.8 ± 0.04	0.8 ± 0.05	0.3 ± 0.01	0.2 ± 0.02

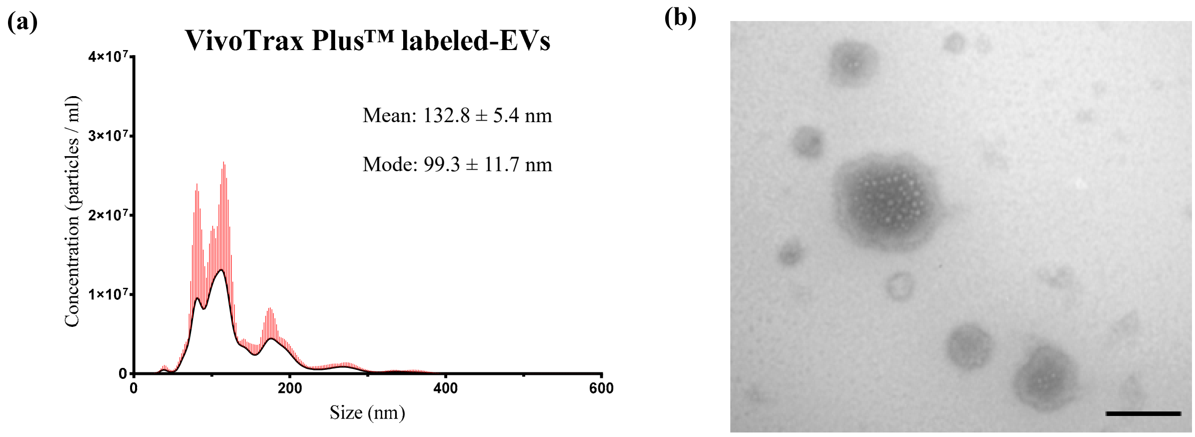

Supplementary Figure S4. (a) Nanoparticle Tracking Analysis (NTA) showing the representative size distribution of the isolated EV population, consistent with the expected size range of small extracellular vesicles. (b) Representative TEM image (56000x magnification) of EVs isolated from VivoTrax Plus-treated ASCs. No electron-dense NPs were visible within membrane-bound vesicles, supporting the absence of NPs into the isolated 30–150 nm EV fraction. Scale bar: 100 nm.

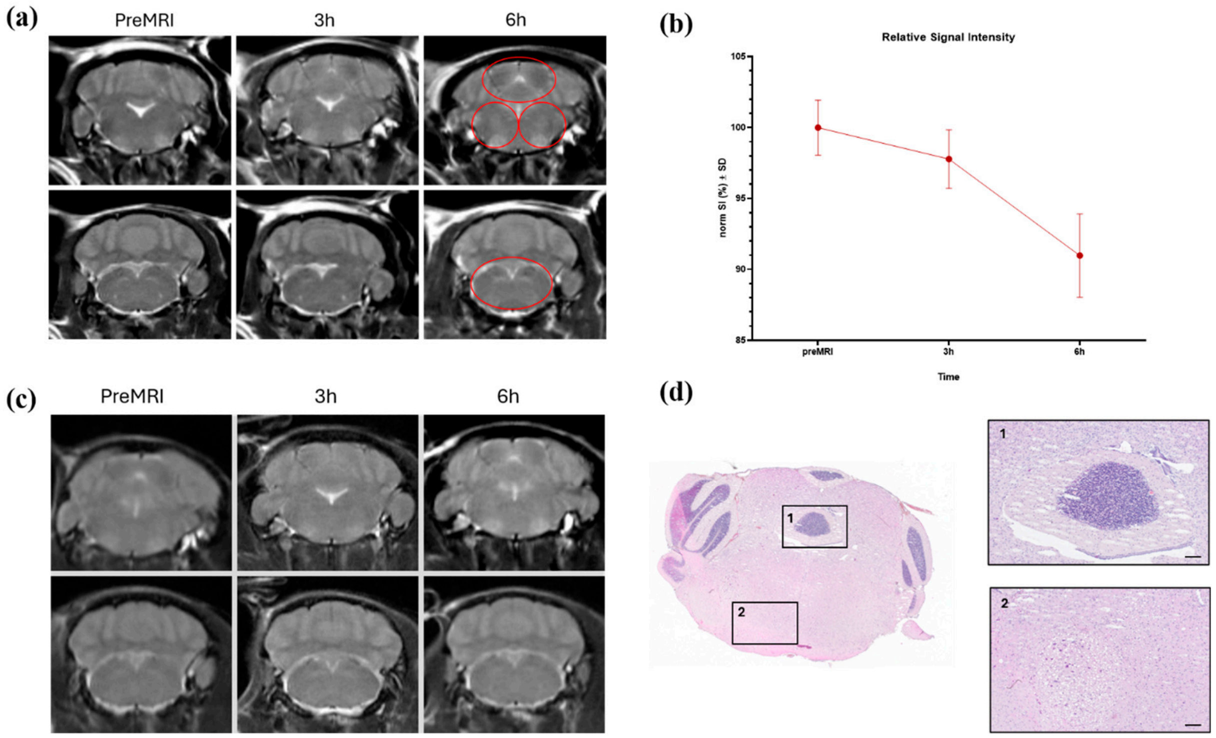

Supplementary Figure S5. (a) Representative T2-weighted MR images from an additional SOD1(G93A) mouse showing preMRI (baseline), 3 h and 6 h after intranasal administration of EVs labeled with VivoTrax. The red circle marked the area in which a hypointense spots is present. (b) Semi-quantitative analysis of normalized whole-brain signal intensity in the three treated animals (mean ± SD). Signal intensity was normalized to the ventricular cerebrospinal fluid signal and averaged over three consecutive coronal slices. (c) Representative T2-weighted MR images from a control SOD1(G93A) mouse receiving unlabeled EVs. (d) Representative Prussian Blue staining of the corresponding control brain, showing the absence of iron-positive staining the lesioned area. The images show the coronal mouse brain slice (10x magnification, scale bar: 100 μm).
